# Theta variation and spatiotemporal scaling along the septotemporal axis of the hippocampus

**DOI:** 10.3389/fnsys.2015.00037

**Published:** 2015-03-16

**Authors:** Lauren L. Long, Jamie G. Bunce, James J. Chrobak

**Affiliations:** ^1^Behavioral Neuroscience Division, Department of Psychology, University of ConnecticutStorrs, CT, USA; ^2^Neural Systems Lab, Department of Health Sciences, Boston UniversityBoston, MA, USA

**Keywords:** dorsoventral axis, septotemporal axis, theta oscillations, locomotor activity, hippocampus, sensorimotor integration, entorhinal cortex

## Abstract

Hippocampal theta has been related to locomotor speed, attention, anxiety, sensorimotor integration and memory among other emergent phenomena. One difficulty in understanding the function of theta is that the hippocampus (HPC) modulates voluntary behavior at the same time that it processes sensory input. Both functions are correlated with characteristic changes in theta indices. The current review highlights a series of studies examining theta local field potential (LFP) signals across the septotemporal or longitudinal axis of the HPC. While the theta signal is coherent throughout the entirety of the HPC, the amplitude, but not the frequency, of theta varies significantly across its three-dimensional expanse. We suggest that the theta signal offers a rich vein of information about how distributed neuronal ensembles support emergent function. Further, we speculate that emergent function across the long axis varies with respect to spatiotemporal scale. Thus, septal HPC processes details of the proximal spatiotemporal environment while more temporal aspects process larger spaces and wider time-scales. The degree to which emergent functions are supported by the synchronization of theta across the septotemporal axis is an open question. Our working model is that theta synchrony serves to bind ensembles representing varying resolutions of spatiotemporal information at interdependent septotemporal areas of the HPC. Such synchrony and cooperative interactions along the septotemporal axis likely support memory formation and subsequent consolidation and retrieval.

## Introduction

You remember the last moment of experience, the last few moments and a variable stream of experience that can extend minutes and hours into the past. Mammals, whether finding food or avoiding becoming food, can remember paths and events that vary in spatiotemporal scale. Thus, you are reading this manuscript on-line, coffee in hand, having sat down at your office computer ten to twenty minutes ago, after having a tense discussion with a colleague in the hallway. Experimental analyses of neurobiological correlates tend to focus on the instantaneous response of the nervous system to sensory and motor events. On the other hand, memory for events occurring over time periods extending minutes, hours or days are dependent upon hippocampal neurobiology. We speculate that emergent function across the long axis varies with respect to spatiotemporal scale. Thus, the septal hippocampus (HPC) represents details of the immediate sensory environment (e.g., right here, right now and the sequence of words in the last sentence), while the temporal HPC represents larger spatial features and longer temporal contexts (e.g., accessing the manuscript, sitting down in your chair and the tense hallway discussion). The reader is also referred to Komorowski et al. (2013); Evensmoen et al. (2015), as well as Wolbers and Wiener (2014) for related discussions of variation across the long axis and variation in spatiotemporal scaling.

Historically, significant emphasis has been placed on examining the functionality of distinct hippocampal (HPC) sub-regions within the tri-synaptic circuit (dentate gyrus (DG) > CA3 > CA1) rather than functional differences across the areal or longitudinal expanse of the HPC. A variety of behavioral studies based on lesion data in rodents and more recently neuroimaging data in humans support functional differentiation of hippocampal circuits along its long axis (Hughes, 1965; Moser et al., 1995; Strange et al., 1999; de Hoz and Martin, 2014; see Bannerman et al., 2004 for reviews see Ta et al., 2012).

How segregate are the circuits and function of different portions of the long axis? The septotemporal axis of the HPC can be subdivided into a septal (dorsal), intermediate and temporal (ventral) portions based on variation in entorhinal inputs (Dolorfo and Amaral, 1998a,b), subcortical projections (Risold and Swanson, 1996) and gene expression (Dong et al., 2009; Fanselow and Dong, 2010; see also Strange et al., 2014 for review). Note the septal HPC in rodents corresponds to the posterior HPC in humans and other primates, while the temporal HPC corresponds to the anterior HPC. The septal portion is generally thought to play a dominant role in spatial information processing, while the temporal portion a greater role in emotion/motivation (see Bannerman et al., 2004 or Fanselow and Dong, 2010 for reviews). Based on subcortical projections to the lateral septum and relays to hypothalamic nuclei, Risold and Swanson (1996) suggested that the septal, intermediate and temporal HPC were differentially involved in guiding different aspects of motivated behavior, ongoing spatial navigation, social and reproductive and ingestive behavior, respectively. While there is clear anatomical and functional differentiation across the long axis, the details of the differences and under what conditions, if ever, is there cooperative interactions across the long axis are open questions.

The theta rhythm is a 6–12 Hz oscillation in the local field potential (LFP) signal that is generated by synchronous synaptic inputs bombarding the somatodendritic field of HPC and entorhinal cortical neurons. The elegant laminar organization of somatodendritic fields within the HPC and the laminar organization of axonal inputs provides a high degree of spatial discrimination to HPC LFP signals. This unique window has in fact helped define temporal structure (e.g., theta, gamma, sharp wave and high-frequency rhythms) in ensemble organization within the brain (Buzsáki, 2006). At the micro-level, the theta rhythm allows for the integration and segregation of individual neuronal elements into distributed cell assemblies (Buzsáki and Chrobak, 1995). At a more macro-level, analysis of areal and laminar variation in the theta LFP signal reveals functional connectivity and emergent function in a manner similar to analyses of electroencephalogric (EEG) and blood-oxygen-level-dependent (BOLD) signals. Thus synchrony in theta phase across regions likely links brain networks into ensemble interactions supporting emergent function (e.g., Remondes and Wilson, 2013). Our laboratory has been examining septotemporal variation in the theta, as well as the gamma signal with respect to self-motion, novelty and experience (Sabolek et al., 2009; Hinman et al., 2011, 2013; Penley et al., 2012, 2013; Long et al., 2014a,b). These studies illustrate septotemporal variation in theta amplitude and frequency in relation to sensorimotor action and experience (e.g., locomotor speed/acceleration), the current sensory environment as well as past experience.

## Hippocampal-Entorhinal Anatomy and Interactions

**Figure 1:**
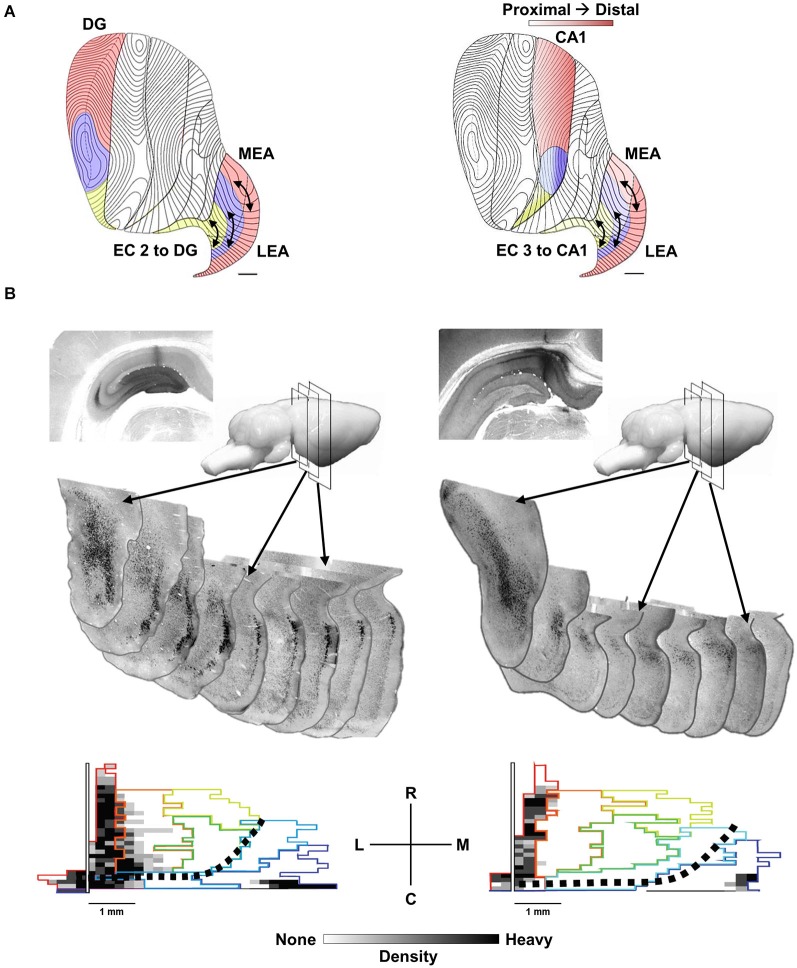
**The topography of EC to HPC projections. (A)** Distinct areas of the provide afferents to the septal 50% of the EC (red), the midseptotemporal 25% (blue) and the temporal 25% (yellow) for DG (left) and CA1 (right). EC projections from layer 2 to the DG and CA3 as well as layer 3 to CA1 exhibit a similar topography. Bands or zones of neurons across the entire rostrocaudal extent of the EC innervate progressively more temporal DG, CA3 and CA1 neurons starting from the caudolateral extreme of the EC toward the more medial aspects of the EC. **(B, left)** cholera toxin b (CTB) injection in septal DG labels rostrocaudal extent of the lateral band (subjacent rhinal sulcus) of EC. Sequential coronal EC sections illustrate labeled (black) EC layer 2 neurons. **(B, bottom left)** Note labeling along rhinal sulcus (left side of map) and caudal extremes (bottom edge). Black dotted line indicates division of traditional medial (bottom right) and lateral (top left) EC. **(B, right)** CTB injection in septal CA1 labels neurons in EC layer 3 with a similar topographic distribution within the lateral band of the EC.

The basic anatomy and physiology of the HPC is highly conserved across mammals and a number of excellent reviews describe the details of this organization (Amaral and Witter, 1989; Lavenex and Amaral, 2000; Strange et al., 2014). We emphasize three features of this anatomy. First as noted above, the HPC has a highly laminar organization that allows for insight into the temporal organization of synchronous synaptic input. Second, there is a topographic organization of entorhinal cortex (EC) inputs to the HPC that maps rostrocaudal-oriented bands or strips of EC neurons to different septotemporal levels of the HPC (Dolorfo and Amaral, 1998a; Chrobak and Amaral, 2007). Third, intrinsic entorhinal associational connections within the bands have the potential to integrate multimodal associative inputs (e.g., visuospatial, auditory, olfactory, self-motion) distributed across the rostrocaudal (anterior-posterior) extent of the EC bands (see Figure 1; Insausti et al., 1987; Witter et al., 1989; Suzuki and Amaral, 1994a; Burwell and Amaral, 1998; Dolorfo and Amaral, 1998a,b; Burwell, 2000; Lavenex and Amaral, 2000; Chrobak and Amaral, 2007; Kerr et al., 2007).

The HPC has a highly laminar organization that allows for insight into the temporal organization of synchronous synaptic input. The principal cell fields including the CA1 and CA3 pyramidal neurons and dentate granule cells as well as associated GABAergic basket cells are densely packed in soldier-like fashion creating the distinctive curvilinear cell layers of region CA1 and CA3 and the sharp-V shape of the granule cell layer (DG). The dendritic fields of these neurons are arranged in relatively narrow tangents oriented roughly ninety-degree from cell layers. The intrinsic intra-hippocampal connections (e.g., mossy cell input to the granule cells, granule cell input to CA3 and CA3 input to CA1) synapse in ordered fashion along the length of the dendritic field of their targets. Similarly, EC layer 2 input to the DG and CA3 and EC layer 3 input to CA1 synapse in an ordered fashion at different somatodendritic locations from the intrahippocampal inputs (see Amaral and Witter, 1989 for detailed description). This ordered architecture allows for spatially unique current flow profiles and isolation of laminar specific changes in LFP signals (see Bragin et al., 1995; Csicsvari et al., 2003; Montgomery et al., 2009).

As noted the septotemporal axis of the HPC can be subdivided into a septal (dorsal), intermediate and temporal (ventral) portions based minimally on variation in entorhinal inputs (Dolorfo and Amaral, 1998a). Neurons within a rostrocaudal band along the dorsolateral and caudal edge of the EC, subjacent the rhinal sulcus except at the most caudal extreme, innervate the septal HPC. Neurons within rostrocaudal bands more medially innervate progressively more temporal aspects of the HPC (Steward and Scoville, 1976; Wyss, 1981; Ruth et al., 1988; Witter et al., 1989; Dolorfo and Amaral, 1998a; see Canto et al., 2008 for review). This is true for EC layer 2 projections to the DG and CA3 as well as layer 3 projections to CA1 (see Figure 1A). It is important to note that the rostrocaudal bands or strips are arranged from the most lateral EC to the medial aspect of the EC, but that this mediolateral orientation is not equivalent with the cytoarchitectonic distinctions between the medial (MEC) and lateral (LEC) EC. The rostrocaudal strips are roughly orthogonal to the MEC-LEC boundaries and thus both MEC and LEC neurons contribute significant input to all septotemporal levels of the HPC. Figure 1B illustrates two retrograde-labeling cases where relatively large tracer injections in septal DG (Figure 1B, **left**) and septal CA1 (Figure 1B, **right**) label a relatively narrow sliver of EC neurons that extend across the entire caudolateral boundary of the EC including both MEC and LEC neurons. Dolorfo and Amaral (1998a) indicated that bands while “not entirely segregate”, exhibited “relatively little overlap” and suggested that “different portions of the entorhinal-hippocampal circuit are capable of semiautonomous information processing”.

In addition to describing the topographic organization of rostrocaudally-projecting EC bands to the HPC, Dolorfo and Amaral (1998a) described a rich network of intrinsic associational connections that could link the neurons within each band. Thus, retrograde and anterograde traces injected anywhere along the rostrocaudal extent of each EC band indicated horizontal associational connections across the entire rostrocaudal extent of each band originating from both superficial (layer 2–3) and deep (V-VI) neurons. The reader is referred to the elegant illustrations in Dolorfo and Amaral, 1998a original publications (Dolorfo and Amaral, 1998a,b) as well as Chrobak and Amaral (2007) for a description of the bands in the macaque. Currently, there is limited additional information about the details of connectivity and physiological interaction across the rostrocaudal extent of each band. Thus, how, when and if grid cell regions within the caudal extent of the lateral band in the MEA interact with functional modules located in the more rostral aspects of each band has not been addressed. It is possible that the long-range horizontal connections within the EC serve to temporally orchestrate the discharge of neurons within discrete functional modules, rather than integrate associative information across different functional modules. In contrast to the limited information about integration across the EC, a larger number of studies have highlighted interlaminar and intralaminar interactions within focal regions of the EC including detailed descriptions of the dorsoventral aspect of the caudal MEC.

Neurophysiological analyses have highlighted MEA and LEA differences (Hargreaves et al., 2005; Deshmukh et al., 2010; Knierim et al., 2013), interlaminar (e.g., deep layer 5 influences on superficial layer 3 and layer 2 neurons; Kloosterman et al., 2003; Ma et al., 2008) and focal intralaminar (e.g., layer 2 to layer 2) most prominently in the MEA (Beed et al., 2010, see Burgalossi and Brecht, 2014 for review). A general finding is that there is greater connectivity among layer 3 and layer 5 neurons than layer 2 pyramidal or stellate cells (see Dhillon and Jones, 2000; Kumar et al., 2007; Ma et al., 2008; Couey et al., 2013; Pastoll et al., 2013). It is important to appreciate that there are two types of principal cells in layer 2, stellate cells and pyramidal neurons, and these distinct types may differentially contribute to local and distant patterns of horizontal, inter-entorhinal connections. Specifically, stellate neurons appear to lack monosynaptic connections with other stellate cells at least locally, although they may contribute to long-range horizontal interactions via disynaptic connections to local and distant GABAergic neurons; the reader is referred to anatomical and physiological descriptions by Klink and Alonso (1997), Buckmaster (2014) and more conceptually to Sasaki et al. (2014) for additional discussion. Burgalossi and Brecht (2014) have recently provided a fairly complete and engaging review highlighting the focal modularity and interconnectivity of the dorsoventral aspect of the caudal MEC. Currently, knowledge of the neurophysiological interactions and detailed anatomical description of inter-entorhinal interactions across the rostrocaudal associational connections is lacking. Nonetheless, we speculate that there is integration of information across the inter-entorhinal bands and that further study is necessary to appreciate these long-range horizontal interactions. On this note, recent findings emphasize direct long-range mono-synaptic interactions of CA1 neurons across the septotemporal axis (Yang et al., 2014) despite the lack of direct focal interactions among CA1 neurons (Deuchars and Thomson, 1996).

The rostrocaudal associational connections are largely orthogonal to the distribution of entorhinal inputs from neocortical associative cortices including the prominent perirhinal cortex, parahippocampal cortex and amygdalar input to the EC (Suzuki and Amaral, 1994; Insausti et al., 1997; Lavenex and Amaral, 2000; Canto et al., 2008; Mohedano-Moriano et al., 2008; Agster and Burwell, 2013). It is thus likely, that these associational connections can integrate across several functional domains defined by both neocortical associative inputs to EC as well as amygdalar inputs. We suggest that inter-entorhinal associative connections within the bands integrate superordinate features of necortical input, such as the spatial features (e.g., proximal-distal) or the time-scale of temporal integration (Giocomo et al., 2007; Hasselmo et al., 2010). In short, the HPC receives distinct sets of EC input that integrate information arriving to different functional domains organized across the rostrocaudal and mediolateral areal axes of the EC.

## The Hippocampal Theta Signal

Hippocampal neurons, entorhinal neurons and multiple subcortical afferents discharge action potentials in phase relation to the theta and theta-related gamma concert. Characteristics of theta and gamma and their relations to emergent function have been elegantly reviewed by multiple authors (Buzsáki et al., 1992; Buzsáki, 2002, 2005; Vertes et al., 2004; Buzsáki and Moser, 2013; Hasselmo and Stern, 2014). Briefly, theta is the relatively slow orchestration of neurons into coordinated “sentences or paragraphs” of information on the time-scale of ~80–200 ms (~5–12 Hz). In contrast, more local gamma rhythmicity is the faster orchestration of neurons into focal ensembles or “letters or words” in theta sentences or paragraphs. Theta and other brain rhythms allow individual neurons to discharge in slow (e.g., theta) and fast (e.g., gamma) temporal relation to other neurons within well-defined as well as rapidly changing ensembles (Buzsáki and Watson, 2012; Dupret et al., 2013).

Mechanistically, theta LFP signals are generated by the summation of relatively synchronous excitatory potentials rhythmically constrained by inhibitory synaptic potentials, impinging on relatively local, but ill-defined regions of somatodendritic space (Green and Arduini, 1954; Petsche et al., 1962; Leung, 1985; Brankack et al., 1993; Bragin et al., 1995; Buzsáki, 2002). The LFP waveform characteristics, such as the frequency and amplitude of the signal depend on the proportional contribution of multiple afferent sources as well as the intrinsic properties of different neurons (Buzsáki et al., 2012). Subtle variation in any one of the inputs, including changes in the timing, can alter synaptic integration and subsequent current flow contributing to the LFP signal. Similarly, Ang et al. (2005) carefully illustrate how subtle temporal variation in the timing of CA3 input to the distal dendrites of CA1 pyramidal cells and dendritic-targeting GABAergic neurons can amplify or suppress the synaptic currents elicited by EC input. Specifically, input from dendritic-targeting GABAergic neurons driven by CA3 input can maximize or suppress intracellular current flow to subsequent EC input within very narrow (20 ms) time windows. The summation of these currents is the origin of extracellular theta LFPs.

Given that virtually all HPC, EC and subcortical afferents discharge in phase relation to the theta dynamic, the LFP signal is relatively coherent throughout the HPC. However, the theta signal varies considerably in shape and amplitude at varying laminar, regional and areal sites in the HPC (see Sabolek et al., 2009). Depending upon the specific location and features of the recording electrodes, researchers can “listen” to signals being generated not by one neuron, but by all the neurons within a three-dimensional range. The elegant anatomical organization of the HPC allows distinctive LFP signals to be “heard” in the same manner in which microphones distributed across an auditorium or stadium could eavesdrop on and isolate the focal generation of auditory signals from orderly arranged sound sources (e.g., rows of “speakers”). Current source density calculations can further isolate these signals (see Csicsvari et al., 2003 for an excellent exemplar) within the HPC, but such are typically limited to two-dimensional analyses and often yield relatively similar results to analyses of the LFP signal.

## Variation in Theta Inputs

**Figure 2:**
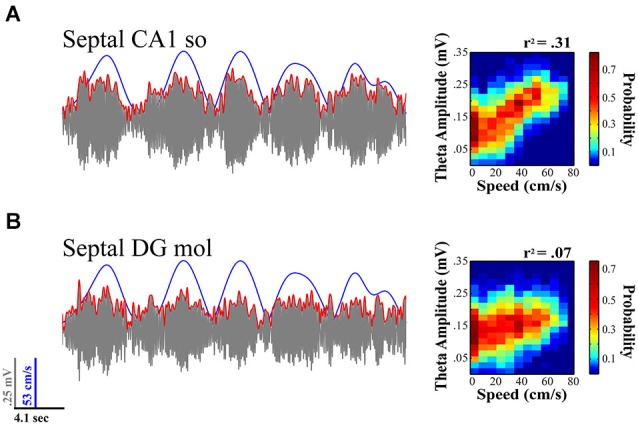
**Theta LFP signal varies across regions and its relationship to locomotor speed within septal HPC**. While fairly coherent within the same septotemporal area, the theta signal varies across lamina within a region (e.g., CA1 stratum radiatum vs. stratum oriens; not shown) and across regions (concurrent CA1 **(A)** vs. DG **(B)** recordings illustrated). The relationship of theta to speed is typically strongest with rats running on a linear track in a highly stereotyped manner and diminishes with multiple aspects of sensorimotor experience (e.g., turns, sensory events, task and memory demands; see text for details). **(A)** Illustration evidences theta variation in relation to speed at concurrently recorded CA1 and **(B)** DG sites for a single 20 s sweep. Theta amplitude to speed traces (left) illustrate relationship over concurrent 5 min recording session while rat navigated on a linear track (see Hinman et al., 2011; Long et al., 2014a). As illustrated, CA1 electrodes typically exhibit a much stronger relation to speed than concurrently recorded DG sites.

The theta signal varies considerably in the three-dimensional expanse of the HPC. The proximodistal and septotemporal topography of the CA3/mossy fiber input to the DG and CA1, as well as the EC into to DG, CA3 and CA1, provide the anatomical substrate for rich variation in the amplitude and synchrony of the synaptic inputs that create the theta LFP signal. The ordered dissonance of inputs from these multiple sources provides variation to the rhythmic drumming determined by the theta frequency, which varies from ~4–12 Hz. The topographic contribution of subcortical inputs including the prominent medial septal projection of GABAergic and cholinergic neurons contributes to the orchestration of the theta signal with regards to both variation in the current (amplitude) and frequency (see Freund and Antal, 1988; Tóth et al., 1993; Lee et al., 1994; Brazhnik and Fox, 1999; Borhegyi et al., 2004; Colom et al., 2005; Manseau et al., 2008). Despite the common understanding that the theta signal is coherent across its laminar and areal axes, the degree of coherence in the amplitude and phase of the signal varies on a moment-to-moment basis reflecting the transient variation in the synchrony of theta generating inputs impinging on the dendritic field structure of distinct populations of neurons. Thus, the signal can vary considerably within a focal region of the septal HPC as illustrated in Figure 2, which illustrates differences in theta amplitude and speed modulation of that amplitude for simultaneously CA1 and DG electrodes.

## Septotemporal Variation in Speed-Theta Indices

**Figure 3:**
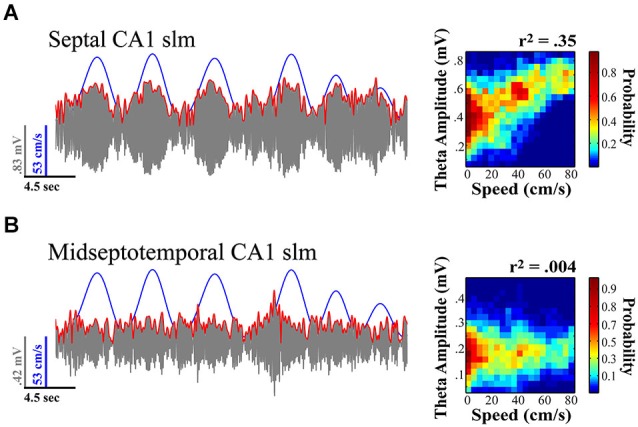
**Theta amplitude varies across the septotemporal axis and the relationship to locomotor speed systematically diminishes with distance from the septal pole of the HPC. (A)** Septal most CA1 sites exhibit the strongest relationship to variation in locomotor speed. **(B)** Sites at the more temporal extremes often exhibit no significant variation in relationship to speed. Notably the relationship of theta to speed is best observed in rats traversing linear tracks (back and forth) and this relationship typically diminishes with turns, task demands, as well as the presentation of sensory events. Illustration indicates example where variation in relation to speed is evident at more septal CA1 stratum lacunosum-moleculare (slm) site as compared to concurrently recorded midseptotemporal site roughly 5mm from septal pole for a single 20 s sweep. Theta amplitude to speed traces (left) illustrate relationship over concurrent 5 min recording session while rat navigated on a linear track (see Hinman et al., 2011; Long et al., 2014a).

Moment-to-moment variation in the power and frequency of the theta signal has been linked to the locomotor speed of the rodent (Vanderwolf, 1969; Teitelbaum and McFarland, 1971; Feder and Ranck, 1973; Whishaw and Vanderwolf, 1973; McFarland et al., 1975). Several laboratories have more recently demonstrated this phenomenon and illustrated that the gamma rhythm also varies as a function of locomotor speed (Rivas et al., 1996; Bouwman et al., 2005; Ahmed and Mehta, 2012; Kemere et al., 2013). While theta varies as a function of locomotor speed, there is a systematic decline in this relationship with distance from the septal pole of the HPC (see Figure 3; Maurer et al., 2005; Hinman et al., 2011; Patel et al., 2012; Long et al., 2014a). This finding coupled with observations that there is an increase in the size of hippocampal places fields across the long axis (Jung et al., 1994; Kjelstrup et al., 2008) support the notion that septal HPC may be encoding the fine details of spatial position while the temporal HPC is tracking larger regions of space (but see, Keinath et al., 2014). We speculate that the significance of the speed-theta relationship represents the flow of sensory input across hippocampal circuitry and it appears that speed “synchronizes” HPC circuits. The faster the animal moves, the faster transitions that need to be made, suggesting that as the animal increases its speed, it has to process incoming information on shorter timescales in order to organize this information into something meaningful. Alterations in the strength and “scaling” in the speed-theta relationship across the septotemporal axis of the HPC could be reflective of HPC processing with high speed-theta relationships indicating efficient processing on shorter time-scales (septal HPC), whereas low speed-theta relationships indicating processing on longer time-scales (temporal HPC). In both cases, the speed-theta dynamic can provide clues with regards to network uncertainty and the subsequent stability of the system as it relates to the predictability of future events (Lisman and Redish, 2009). In support of these ideas, Terrazas et al., 2005 attenuated self-motion signals and suggests that the speed-signal is crucial for determining the scaling of place representation. When motion signals are attenuated, the HPC responds as if the animal were moving slower through space, traveling across a smaller distance and subsequently making place fields larger. These outcomes were consistent with reductions in neuronal firing rates as well as theta amplitude gain in response to locomotor speed. Consequently, these results may suggest that alterations in spatial scale across the long axis of the HPC could potentially be described by systematic variation in the gain of a motion signal (Maurer et al., 2005; Terrazas et al., 2005; McNaughton et al., 2006).

**Figure 4:**
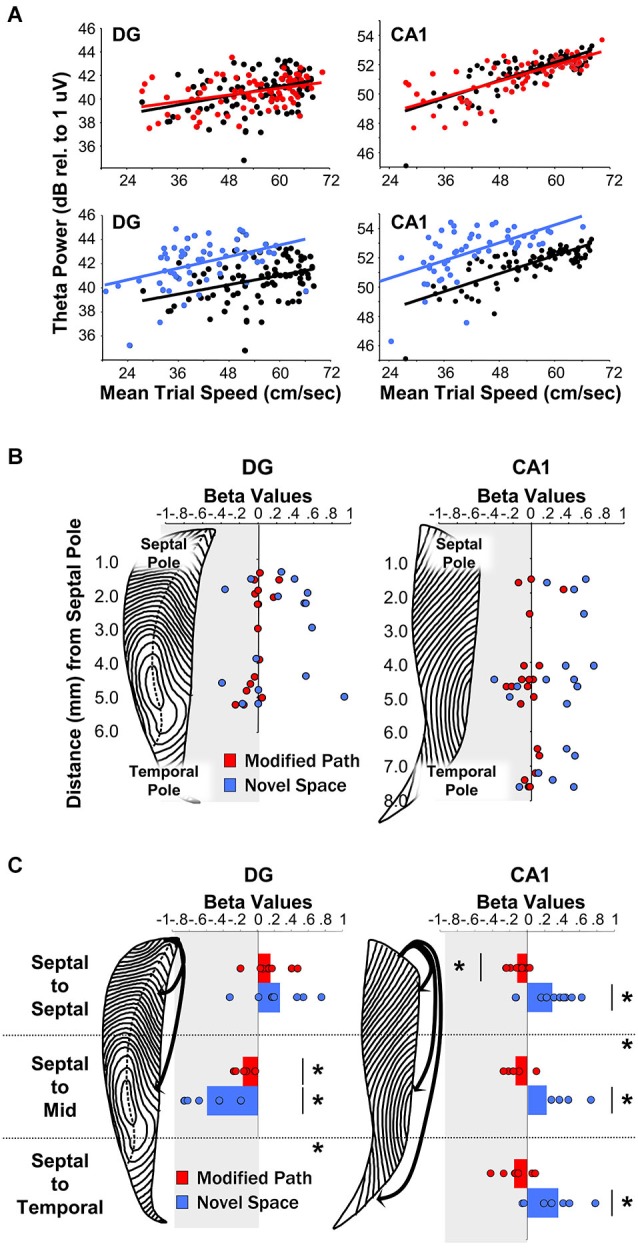
**Theta LFP increases irrespective of locomotor speed during exposure to a novel spatial environment**. (Figures adapted from Penley et al. (2013), *Frontiers in Neuroscience*). **(A)** Baseline theta power values in CA1 and the DG as a function of the average trial speed for a single animal in the familiar condition (black), on the modified path (red), and in the novel space (blue). **(B)** Distribution of β-values (standardized regression coefficient) from individual electrode sites within the DG (left) and CA1 (right) at different septotemporal positions. Points indicate changes in the power of theta power on the modified path (red) and novel space (blue) from the familiar condition. **(C)** β-values for coherence across all electrode pairs within septal, across septal, and mid-septotemporal sites and across septal and temporal sites comparing changes in the modified path (red) and novel space (blue) from the familiar condition for DG pairs (right column) and CA1 pairs (left column). For categorical variables (familiar vs. novel space), β-values indicate changes in theta power and coherence independent of alterations in locomotor speed (see Hinman et al., 2011; Penley et al., 2013; see also Long et al., 2014a/b for additional information with regards to β-values for continuous variables).

**Figure 5:**
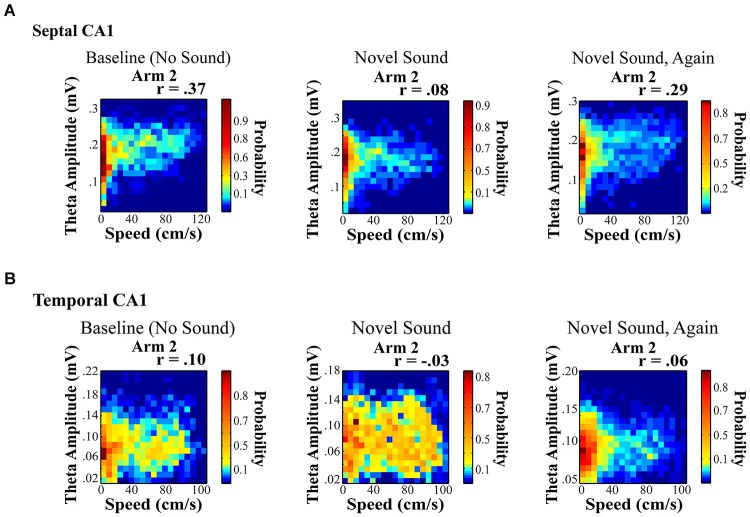
**Novel sound presentation decreases the relationship between theta amplitude and locomotor speed in a location specific manner**. Rats were trained to run on a rectangular maze for a food reward. **(A)** and **(B)** illustrate a significant decrease in the relationship between speed and theta for septal **(A)** and temporal **(B)** electrodes on Arm 2, which was the arm that was in closest proximity to the sound source (see Long et al., 2014b for additional details). Surprisingly, we observed a unique reduction in the speed to theta relationship only on the arm nearest the sound source with a habituation of this decreased slope across repeated sound exposures (right).

In contrast to theta amplitude, the frequency of theta is a relatively fixed phenomenon across the length of the septotemporal axis (Hinman et al., 2011; Patel et al., 2012). The frequency across the entire HPC as well as the EC is likely a consequent of the phase related firing of subcortical inputs including those from supramammilary nucleus and the medial septum (MS; Freund and Antal, 1988; King et al., 1998; see Mattis et al., 2014 for recent overview). While cholinergic medial septal cells are thought to contribute to alternations in theta amplitude changes (Lee et al., 1994; Buzsáki, 2002), they lack the temporal resolution to contribute to rapid changes in theta power (Zhang et al., 2010; Vandecasteele et al., 2014); whereas cells that participate in theta current generation have the ability to produce theta amplitude changes on a finer temporal scale. Further, with segregate MS neurons projecting to differential septotemporal extents of the HPC, the MS is well suited for synchronizing or desynchronizing the theta rhythm across the longitudinal axis. Our observations indicate that the strongest relationship between locomotor speed and theta amplitude is observed in the most septal CA1 electrodes in rats running in a highly stereotyped manner across a linear track. While it has not necessarily been systematically examined, multiple findings along with our own unpublished observations evidence that locomotor speed does not always account for a significant portion of the variability in theta amplitude (e.g., Montgomery et al., 2009; Gupta et al., 2012; Jeewajee et al., 2013; Schmidt et al., 2013; Long et al., 2014b; see Figures 4, 5). One may suppose that the strength of the relationship increases with experience or only on linear tracks where running behavior becomes more stereotyped (see Jeewajee et al., 2013). On the other hand, we have observed a systematic decrease in theta power and the speed-theta relationship over repeated daily sessions within a day (Hinman et al., 2011). These findings certainly illustrate that the relationship between running speed and the amplitude of the theta signal is not fixed and can vary depending upon a number of key variables.

## Spatial Novelty and Experience Variation

A wealth of evidence links the HPC to novelty detection and neurophysiological signals within the HPC typically habituate or decrease with repeated experience (see Vinogradova, 2001; Nyberg, 2005; Kumaran and Maguire, 2009 for review see Kemere et al., 2013). Our recent findings reveal that rats navigating across a runway in a novel space, as compared to a familiar environment, exhibit an increase in theta power across electrode sites throughout the entire septotemporal extent of the HPC including sites in DG and CA1 (Penley et al., 2013; see Figures 4A/B). Further, there was an increase in theta coherence across septotemporally distant CA1 electrodes although not across DG electrodes (Figure 4C). These findings suggest that environmental novelty synchronizes and engages the entirety of the septotemporal axis to encode novel spatial experience. We suggest that within limits, greater power and coherence reflect a numerically larger, and temporally more precise, network engagement in a common process; in this case, the CA1 network engaged in encoding the features of a novel spatial experience.

In contrast to changes in response to novelty, we have also reported that theta amplitude decreases as a function of experience across repeated trials on a linear track. This phenomenon is prominent at more temporal levels of the HPC, with no habituation observed at septal electrodes (see Figure 6 in Hinman et al., 2011). These data may indicate that the septal HPC is continually engaged by the details of current experience while more temporal levels encoding larger spatial features and longer time spans may habituate when meaningful information about such spatiotemporal features are not relevant or have no necessary significance to ongoing behavior or cognitive demands. It has been observed that theta in the most temporal aspects of the HPC is minimal in amplitude and intermittent in occurrence (Royer et al., 2010). The latter may reflect the significance (or lack thereof) of information concerning larger spatiotemporal phenomenon. Thus, HPC circuits may, or may not, need to maintain information about the experience and spatial context of events that occurred in the past five minutes or hour, if that information is no longer relevant to ongoing cognitive performance or future behavior. In contrast, when navigating for the first time in a new city, novel tourist destination or foraging environment, the spatiotemporal details of both experience and navigation are more relevant to ongoing and future behavior.

Recent data from Patel et al. (2012) investigated a hypothesis first put forth by Lubenov and Siapas, 2009 suggesting there exists a 360° phase shift in the theta wave between septal and temporal HPC sites. Data from Patel et al., 2012 indicate not a 360°, but a 180° phase shift between septotemporal sites. These data are important to consider with regards to experience and behavior dependent alterations in septotemporal theta indices. Overall, these data suggest that distributed groups of neurons can assimilate or segregate how multimodal neocortical sensory features can be perceptually integrated or consolidated into memories across the septotemporal axis of the HPC. Alterations in network interactions may provide a clue into the segregation of information among areal regions of the HPC where changes in septotemporal synchrony may offer insights into when distributed networks interact. We hypothesize that this phase shift is not a simple product of fairly fixed anatomical constraints but degree of environmental familiarity and task demands may modify the aforementioned phenomenon suggesting that environmental requirements may serve to segregate or enhance HPC networks along the long axis. Further, we propose that theta coordination across the long axis reflects a shifting dynamic between CA3 and EC afferents. Such coordination allows subsets of CA3 neurons to discharge at earlier phases of theta relative to EC neurons. This shift in phase may reflect the efficacy of EC synaptic inputs. Slight alterations in theta frequency representing the timing of inputs can bias the response of CA1 neurons to either CA3 or EC input (Ang et al., 2005). Similarly, Hasselmo et al. (2002), Hasselmo (2005) describe theta as providing bias to different synaptic inputs at different phases of each theta cycle (Hasselmo et al., 2002; Hasselmo, 2005). Such a biasing mechanism could allow for the preferential encoding of new representations (e.g., novelty; EC input dominating) or under conditions of familiarly, largely ignoring EC input and responding to CA3 inputs. Although speculative, more likely than not environmental variables will bias synaptic inputs ultimately resulting in differential degrees of septotemporal theta wave phase shifts.

## Sensory Novelty

Hippocampal neurophysiological indices typically increase in relation to any novel stimulus within various stimulus modalities including: auditory cues (Redding, 1967; Parmeggiani and Rapisarda, 1969; Parmeggiani et al., 1982), textures (Itskov et al., 2011), odors (Wiebe and Stäubli, 1999, 2001; Wood et al., 1999; Martin et al., 2007; Komorowski et al., 2009; Gourévitch et al., 2010) and gustatory cues (Ho et al., 2011). Auditory stimuli present an easily modifiable signal that affords considerable novel and temporal control. In a recent study, we were interested in whether the presentation of a novel sensory stimulus could alter theta indices in a manner similar to navigation in a novel spatial environment. Our findings were different from what might be expected. First, the presentation of a novel acoustic stimulus in a familiar environment modified the speed to theta amplitude relationship in a location specific manner. Second, the novel sound decreased the slope and r-square of the speed-theta relationship, which habituated (returned to baseline) across repeated sound exposures (Figure 5; see also Long et al., 2014b). A few details on these results are noteworthy as they may offer insight into the dynamics and variation of the hippocampal theta signal. Briefly, rats were trained to run on a rectangular maze for a food reward. Once baseline recordings (no sound) were obtained from well-trained rats in a highly familiar spatial environment, multiple recordings sessions were collected in the presence of a chronic sound stimulus presented nearest one arm of the rectangular maze (see Long et al., 2014b for additional details). The only observed change was a decrease in the slope and r-square of the locomotor speed to theta amplitude relationship during a single ten minute run session (Figure 5), which subsequently habituated across repeated sound exposures.

The effects of a novel acoustic stimulus were strikingly different from exposure to a novel spatial environment. Novel space dramatically increased theta power, which may result from an overall novelty related increase in one or more modulatory inputs (e.g., cholinergic, noradrenergic). The novel acoustic stimuli exerted a fundamentally distinct effect inducing a sharp reduction in the slope and r-square of the speed to theta amplitude relationship in a location specific manner, despite the omnipresence of the acoustic stimulus. Ongoing studies are currently exploring the location specific changes in the theta amplitude to locomotor speed relationship and how this phenomenon varies across the septotemporal axis, as recent data has suggested that the LFP can encode spatial information as robustly as single units (Agarwal et al., 2014).

## Cognition and the Hippocampal Formation

Network activation as measured by LFP theta signals in the HPC can be used as a tool to better understand moment-by-moment dynamics across the septotemporal axis of the HPC. Similar to analysis of variations in the blood-oxygen-dependent (BOLD) signal used in functional neuroimaging (Logothetis and Wandell, 2004; Law et al., 2005), detailed analysis of theta signal reveals the engagement of distributed neural circuits in relation to ongoing sensorimotor experience as well as cognitive operations in both humans (Kahana et al., 1999; Fell et al., 2001; Burgess et al., 2002; Sederberg et al., 2003; Ekstrom et al., 2005; Canolty et al., 2006; Knake et al., 2007) and non-human animals (Berry et al., 1978; Winson, 1978; Hasselmo et al., 2002; Wyble et al., 2004; Asaka et al., 2005; Buzsáki, 2005; Hasselmo, 2005; Kay, 2005; Jeewajee et al., 2008; Kheirbek et al., 2013).

Our review illustrates that there is significant variation in the theta LFP signal across the septotemporal axis and that characteristics of that signal vary differentially with respect to locomotor speed and experience. Importantly habituation or repeated exposure to the same task in the same environment decreases the theta signal most prominently at progressively more temporal HPC sites (Hinman et al., 2011) with no significant habituation at the most septal HPC sites. The latter is consistent with the noted intermittency in hippocampal theta reported by Royer et al. (2010). It appears the mechanisms that generate theta in the more temporal aspects of the HPC diminish, and/or the window for synaptic integration is larger (Marcelin et al., 2012) upon repeated exposure to the same sensory environment or repetition of voluntary motor activity.

In addition to changes in theta power, changes in theta synchrony (coherence) can be observed across the septotemporal axis. Novel spatial environments increase theta coherence across the long axis of CA1. Thus, changes in theta synchrony vary predictably with environmental spatial conditions (Penley et al., 2012, 2013) as well as alterations in the pattern of voluntary motor activity (Hinman et al., 2011; Long et al., 2014a). While these findings highlight alterations in the theta LFP with respect to novel spatial phenomenon, theta also reflects aspects of motor performance. Thus, changes in theta amplitude can precede locomotor activity by hundreds of milliseconds, perhaps indicating a role for HPC circuits in anticipating or contributing to the selection of future movements (Wyble et al., 2004; Long et al., 2014a). Additionally, Vanderwolf (1971) and Whishaw (1972) demonstrated that cessation of theta activity initiated by termination of locomotion is associated with an onset of small-amplitude irregular activity (see also Gray and Ball, 1970; Kimsey et al., 1974). We have also reported a sharp reduction in theta amplitude during deceleration that generally occurs at the termination of locomotion (Long et al., 2014a). This observation is consistent with that presented by Wyble et al. (2004) where a sharp decrease in theta power (240–400 ms) precedes the cessation of locomotor activity. Further, Bland and Oddie (2001) suggest that theta as manifested by hippocampal and associated structures functions to provide “voluntary motor systems with continually updated feedback on their performance relative to changing environmental (sensory) conditions”. This general theoretical framework is supported by the underlying anatomy of hippocampal circuits that link multimodal associative cortices to ventral basal ganglia circuits (Mogenson et al., 1980; Sesack and Grace, 2010; Aggleton, 2012).

These data suggest that theta amplitude could be more related to future behavioral performance. A number of studies have investigated the relation of hippocampal unit spiking to past and/or future behavioral states. The spatial path represented during spiking activity witnessed in each theta cycle has been the focus of most of these studies (Dragoi and Buzsáki, 2006; Foster and Wilson, 2007; Johnson and Redish, 2007; Maurer et al., 2012) and suggests that hippocampal place cell activity is, at times, more reflective of future behaviors (Frank et al., 2000; Wood et al., 2000; Ferbinteanu and Shapiro, 2003; Ji and Wilson, 2008). In this vein, Schmidt-Hieber and Häusser (2013) demonstrate that theta membrane potential oscillations in medial EC preceded the onset of running, in some cases by more than one second. Similarly, Gupta et al. (2012) discuss acceleration and deceleration with regards to theta sequences. They demonstrate that as rats accelerate, paths represented are shifted forward in space, whereas during deceleration, paths are shifted backward in space. The authors relate this phenomenon to anticipation of reaching desired locations, where paths shifted backward in space may serve to review current experience (Ji and Wilson, 2008). Thus, the suggestion that hippocampal processing represents only “the here and now” is highly doubtful (Yartsev, 2008). Imaginably, this distinction could be a consequence of variation in the spatiotemporal scale of neuronal representations and diffrences in that scaling across the septotemporal axis. Notably, septal HPC “place” fields are narrowly tuned in the spatial domain and relatively insensitive to motivational variables (hunger, anxiety). In contrast, more temporal neurons have progressively larger place fields and are sensitive to emotional state (Jung et al., 1994; Kjelstrup et al., 2008; Royer et al., 2010). Recent findings also demonstrate “time cells” in the septal HPC (Pastalkova et al., 2008; Kraus et al., 2013) which increase their firing rates during specific seconds across the delay of a delayed conditional discrimination (MacDonald et al., 2011). Thus, the latter may represent the importance of spatiotemporal phenomenon across multiple time-scales.

## Conceptual Framework

The hippocampal formation (HF; includes HPC) supports episodic memory formation in the mammalian brain. A specific characteristic of episodic memory is that it requires the recruitment of numerous different sensory modalities across multiple spatial and temporal scales. Data indicate functional differentiation across the septotemporal axis of the HPC with septal HPC supporting “spatial memory” and temporal HPC “emotional memory”. Does the septotemporal axis of the HPC act as a unitary structure for successful information processing, or can areal domains segregate depending upon cognitive demands? This distinction could arise because of variations in spatiotemporal scale (e.g., “time” cells; MacDonald et al., 2011; Kraus et al., 2013) across the long axis of the HPC as mediated by differences in sub-cortical modulation or variations in specific receptor-activated membrane conductances (Moser and Moser, 1998). These intrinsic septotemporal differences along with anatomical data support a role for oscillations in the binding of relevant, multi-modal information as it relates to episodic events.

With these ideas in mind, high-frequency oscillations (e.g., gamma) reflect “local” network processing and feature binding across brain regions (e.g., primary auditory cortex), while low-frequency oscillations (e.g., cortical beta rhythm) are dynamically entrained across distributed brain areas (e.g., communication of sensory information to HPC). Synchronization of oscillations may serve as a mechanism to transfer and bind information from large-scale network events operating at behavioral time-scales, to fast, local events operating at smaller time-scales—which are needed for synaptic adaptation (Buzsáki, 2006). The consequence of such activity is the integration and combining of events across multiple spatiotemporal scales (Canolty et al., 2010). Each sensory system (e.g., auditory, visual) generates oscillations at particular frequencies (e.g., beta oscillation) in response to relevant input and stimuli (Haenschel et al., 2000; Kay and Beshel, 2010; Cervenka et al., 2013). Given the aforementioned suggestions, it is likely that the HPC encodes and retrieves such sensory information via its own, internally generated oscillations (e.g., theta and gamma) and thus provides a framework for how the HPC gains access to episodic information spanning multiple modalities and spatiotemporal scales. In the current review, we emphasize the role of septotemporal theta indices with respect to recent familiar and novel spatial, as well as sensory experience. We indicate that septotemporal variation in theta dynamics may arise as a consequence of differences in the representation of spatiotemporal scale. These implications have far reaching consequences with regards to hippocampal processing and computation.

### Summary

Here, we indicate that HPC theta oscillations can be related to locomotion, sensory and spatial novelty as well as recent experience. Data from our lab and others support a role for the septotemporal axis in the processing of spatial and sensory novelty—both exerting fundamentally different effects on theta dynamics. Further, novelty induced alterations in theta indices habituated across repeated exposure to a novel environment and auditory stimulus. These results are not inconsistent with reports evidencing habituation of novel auditory stimuli in auditory cortex (Haenschel et al., 2000). We speculate that factors such as time on maze and the familiarity of the experience differentially engage septotemporal circuits, supporting the idea that spatiotemporal scale is differentially represented across the long axis. Thus, septal HPC may be continually engaged by the details of the experience (as indicated by constantly high theta power), while more temporal aspects of the hippocampus, become “bored” and disengage more readily when meaningful information about such spatiotemporal features are not relevant to current task demands (as indicated by theta amplitude decrement). These data support the integration of events (e.g., episodic memory) across modalities—as represented by HPC afferents arising from EC, which funnels multi-modal sensory information to HPC and spatiotemporal trajectories—as indicated by alterations in the representation of space and time across the long axis.

## Conflict of Interest Statement

The authors declare that the research was conducted in the absence of any commercial or financial relationships that could be construed as a potential conflict of interest.
